# Utilization of Organs from Hepatitis C-Antibody-Positive or RNA-Positive Donors in Kidney Transplantation: A Single-Center Retrospective Analysis of Outcomes and Safety

**DOI:** 10.3390/jcm14082653

**Published:** 2025-04-12

**Authors:** Lara Ploeger, Philipp Luetke Elshoff, Birgit Kortus-Goetze, Joachim Hoyer, Martin Russwurm, Johannes Wild

**Affiliations:** 1 Department of Internal Medicine and Nephrology, University Hospital Marburg, 35043 Marburg, Germany; lara.ploeger@uk-gm.de (L.P.);; 2 Pharmacological Institute, University Marburg, 35043 Marburg, Germany

**Keywords:** kidney transplantation, hepatitis C, donor-derived infections, organ allocation

## Abstract

**Background/Objectives**: The shortage of donor organs in transplant medicine remains a challenge. Kidney transplantation from Hepatitis C (HCV)-positive donors to HCV-negative recipients expands the donor pool. Limited data suggest this approach as safe when combined with modern antiviral therapies. This study evaluates the safety of such transplantations in terms of viral transmission and graft function. **Methods**: A retrospective analysis of 205 kidney transplantations at the University Medical Center Marburg (January 2017–January 2024) was conducted. Eight recipients received kidneys from HCV-antibody-positive (HCV-Antibody+) and RNA-negative donors (HCV-RNA−), and five received kidneys from HCV-RNA-positive (HCV-RNA+) donors. Recipient demographics, donor factors, and transplantation parameters were analyzed. Repeated virological surveillance as well as graft function and complications were assessed within the first year after transplantation. **Results**: HCV-RNA+ donor recipients received Glecaprevir/Pibrentasvir for three months starting immediately at transplantation, while HCV-Antibody+ and HCV-RNA− donor recipients did not receive antiviral therapy. After 12 months, both groups exhibited comparable graft function (serum creatinine: HCV-Antibody+/RNA− 1.3 ± 0.4 mg/dL vs. HCV-RNA+ 1.8 ± 0.5 mg/dL, *p* = 0.6) without proteinuria. No hepatic complications or significant inflammation occurred. No HCV-RNA was detected in any patient at any time under the selected treatment regimen. **Conclusions**: This single-center study supports the safety of kidney transplantation from HCV-positive donors. Preemptive Glecaprevir/Pibrentasvir therapy effectively prevents HCV transmission, offering a viable option to expand the donor pool.

## 1. Introduction

Chronic kidney disease affects 10% of the global population [1], and its prevalence is rising [2]. Progressive decline in kidney function ultimately necessitates the initiation of kidney replacement therapy (KRT), through either dialysis or kidney transplantation. Kidney transplantation is considered the treatment of choice for most patients, since long-term survival for patients undergoing kidney transplantation is significantly better compared with patients who remain on dialysis [3,4]. Organ shortage is a major problem as it affects all patients without a suitable living donor. There are geographical differences, but waiting times for a kidney are long and can be up to 10 years [5]. In Germany, the average waiting time is 8.9 years [6]. The mortality of patients on the waitlist is high: approximately 50% of transplant candidates die before receiving a transplant organ [5,7].

Taking the rising prevalence of chronic kidney disease and scarcity of organs into consideration, transplant physicians are looking for ways to increase the availability of organs. In the past, organs from patients with active infectious diseases, such as Hepatitis C, were discarded due to the risk of infection [8]. The Hepatitis C virus (HCV) is a single-stranded RNA virus [9] that is transmitted parenterally via contact with contaminated blood. Diagnosis is based on the detection of anti-HCV antibodies. However, a positive antibody test does not allow a distinction between active disease or treated/resolved disease. Nucleic acid testing (NAT) is used to prove active HCV replication and later to evaluate treatment effects [10]. It is important to note that HCV may persist in the liver of HCV-Antibody+ patients, even when HCV-RNA is undetectable in blood samples. This suggests that HCV-Antibody+/RNA- patients may either have a resolved infection or an HCV infection without detectable viremia, both of which are considered prognostically favorable states [11]. The risk for HCV transmission is high in cases when organs from HCV-RNA+ viremic donors are transplanted into a recipient without HCV.

However, with safe and effective HCV treatment options available, the acceptance of such organs is rising [8]. Until 2011, the standard therapy regimen was based on interferon and ribavirin. When employing this therapy, sustained virological response (SVR) was achieved in 50% of cases [9]. With the introduction of direct antiviral agents (DAAs), medications with a significantly better adverse-effect profile than interferon-containing regimens are available [10]. The duration of therapy is significantly shorter, and SVR is achieved in up to 95% of cases [12]. In the context of kidney transplantation involving HCV-RNA+ donors and HCV-RNA- recipients, no consensus exists regarding the optimal timing for initiating antiviral therapy. The current literature primarily describes commencing therapy only after HCV-RNA is detected in the recipient post-transplantation [13]. To date, there are very limited published real-life data on the use of preemptive antiviral therapy initiated immediately following transplantation [14]. We therefore aimed to retrospectively evaluate the experience at our center, where direct preemptive therapy with Glecaprevir/Pibrentasvir is initiated for a duration of three months in kidney recipients from HCV-RNA+ donors. Moreover, there is a lack of empirical evidence regarding the clinical relevance of isolated HCV antibody detection in deceased kidney donors [15]. To address this gap, we compared outcomes in recipients of kidneys from donors with isolated HCV antibody detection to those from donors with both HCV antibody and RNA detection.

## 2. Materials and Methods

We conducted a retrospective study including all kidney transplantations at the University Medical Center Marburg from 01/2017 to 01/2024 (*n* = 205). We identified 8 patients who received a kidney from an HCV-Antibody+ but HCV-RNA- deceased donor (reflecting resolved infection at the time of organ donation) as well as 5 patients who received a kidney from an HCV-RNA+ donor (reflecting active infection at the time of organ donation). The center’s internal protocol mandates that all patients added to the waiting list receive both verbal and written information in advance regarding the possibility of receiving a kidney from an HCV-RNA+ donor. Additionally, the following patients were generally excluded from receiving organs from HCV-RNA+ donors: highly immunized patients, those with focal segmental glomerulosclerosis as the underlying disease, and patients with a known allergy to Tacrolimus. Patients who were HCV-Antibody+ but HCV-RNA−, as well as those with a history of Hepatitis B infection (due to the potential for reactivation under antiviral therapy), were also excluded. Furthermore, patients with persistent elevation of liver enzymes (more than two times the upper normal limit), a history of liver disease, or polycystic kidney disease with concomitant liver cysts were not considered for organ transplants from HCV-RNA+ donors. Additionally, patients unable to provide informed consent due to a lack of decision-making capacity and those requiring mandatory premedication with drugs known to interact with antiviral therapy (such as Phenytoin, Carbamazepine, Primidone, or Bosentan) were excluded from receiving kidney transplants from HCV-RNA+ donors. There were no such restrictions for organs from HCV-Antibody+/RNA− donors.

We included these 13 patients in our analysis and compared clinical courses from patients receiving organs from HCV-Antibody+/RNA− vs. HCV-RNA+ donors. We obtained recipient-related data regarding age, sex, start of dialysis, cause of end-stage renal disease, and dialysis modality. Moreover, we assessed donor-related factors and transplant-related factors as ischemia time or mismatches. All blood parameters were measured in a certified clinical laboratory as part of the diagnostic routine. The study was approved by the institutional ethics review board (Approval Number 24-90RS, Ethics Committee Philipps University Marburg, Germany). All procedures were conducted in accordance with the Declaration of Helsinki. 

Statistical analyses were conducted using GraphPad Prism software (version 10; GraphPad Software Inc., San Diego, CA, USA). Data are presented either as individual values or as the mean ± standard error of the mean (S.E.M). Normality of the data was assessed using the D’Agostino and Pearson test, and outliers were identified using the ROUT method with a Q value of 1% (maximum desired False Discovery Rate). Descriptive statistics for comparisons of key characteristics between recipients or donors of HCV-Antibody+ kidneys vs. HCV-RNA+ kidneys are reported as medians with standard deviations (SDs) or as absolute numbers with corresponding percentages. For continuous variable comparisons, we applied the Mann–Whitney U test or *t*-test, depending on the distribution, while categorical variables were assessed using Fisher’s exact test. Additionally, mixed-effects analysis with multiple comparisons as well as multiple *t*-tests were employed where applicable. Statistical significance was defined as a *p*-value of <0.05, with significance levels indicated as follows: * *p*  <  0.05, ** *p*  <  0.01, and *** *p*  <  0.001.

## 3. Results

A total of 13 kidney transplant recipients were included in the study, with 8 patients receiving kidneys from HCV-Antibody+/RNA− donors and 5 patients receiving kidneys from HCV-RNA+ donors (Table 1). The median age at transplant was 52.85 ± 11.4 years for the entire cohort. The median age for the HCV-Antibody+/RNA− group was 48.9 ± 12.5 years, and it was 59.2 ± 4.3 years for the HCV-RNA+ group; the age difference was not statistically significant (*p* = 0.13). Body mass index (BMI) was similar between the groups, with a mean BMI of 26.9 ± 3.1 for the overall cohort. All patients were on hemodialysis prior to kidney transplantation; the duration of hemodialysis prior to transplantation was significantly longer in the HCV-Antibody+/RNA− group (102 ± 27.3 months) compared to the HCV-RNA+ group (44.6 ± 20.0 months, *p* = 0.003). Residual diuresis, defined as urine output independent of dialysis, was observed in 61.5% of all recipients. Hypertension was present in the majority of recipients (76.9%), and no significant difference was observed between the two groups (*p* = 0.99). Similarly, diabetes mellitus was less common, affecting 15.4% of the cohort, with no significant difference in prevalence between the two groups (*p* = 0.99). The causes of end-stage renal disease were varied, with cystic disease being the most common, accounting for 23% of all cases. Other causes included diabetes, hypertension, congenital anomalies of the kidney and urinary tract, and glomerulonephritis.

Total ischemia time, defined as the duration between organ retrieval and transplantation, was slightly longer in the HCV-RNA+ group (976.2 min ± 419.0) compared to the HCV-Antibody+/RNA− group (750.4 min ± 342.0), although this difference did not reach statistical significance (*p* = 0.35). The mean number of mismatches between donor and recipient was comparable between the two groups, with a value of 3.5 ± 1.2 in the HCV-Antibody+/RNA− group and 3.4 ± 1.0 in the HCV-RNA+ group, showing no significant difference (*p* = 0.76). Cytomegalovirus (CMV) positivity in recipients was observed in 46.2% of all patients, with no significant difference between groups (*p* = 0.99). CMV positivity in donors, however, was more prevalent in the HCV-RNA+ group (80%) compared to the HCV-Antibody+/RNA− group (37.5%), but this difference was also not statistically significant (*p* = 0.27). Following the standard operating procedures of the center, a significant difference was observed in the administration of antiviral treatment with Glecaprevir/Pibrentasvir, which was given for 3 months post-transplant only to patients receiving kidneys from HCV-RNA+ donors. All patients in the HCV-RNA+ group received this treatment (100%), while none of the patients in the HCV-Antibody+/RNA− group were treated (*p* = 0.001). The baseline immunosuppression was administered according to the center’s internal standards (Table 1). Recipients of HCV-Antibody+/RNA− organs without immunization events or preformed antibodies received a combination of Cyclosporin A, Mycophenolate Mofetil, Prednisone, and Basiliximab (when there were three or more mismatches). In pre-immunized patients, Tacrolimus was used instead of Cyclosporin A. Recipients of organs from HCV-RNA+ donors always received Tacrolimus due to Organic Anion Transporting Polypeptide-related drug interactions between Cyclosporin A and Glecaprevir/Pibrentasvir.

Regarding donor characteristics (Table 2), there was no significant difference in the proportion of female donors between the two groups, with five (62.5%) female donors in the HCV-Antibody+ group and two (40%) female donors in the HCV-RNA+ group (*p* = 0.59). The median age at donation was slightly younger in the HCV-Antibody+/RNA− group (44.25 years ± 11.22) compared to the HCV-RNA+ group (56.40 ± 9.72 years), although this difference did not reach statistical significance (*p* = 0.09).

Donor serum creatinine levels were similar between the groups, with a median creatinine level of 1.09 ± 0.68 mg/dL in the HCV-Antibody+ group and 0.86 ± 0.28 mg/dL in the HCV-RNA+ group (*p* = 0.32). In terms of liver function, the total bilirubin levels were slightly higher in the HCV-RNA+ group (0.78 ± 0.56 mg/dL) compared to the HCV-Antibody+ group (0.37 ± 0.15 mg/dL), although this difference was not statistically significant (*p* = 0.13). Similarly, liver enzymes, including aspartate aminotransferase (ASAT) (*p* = 0.42), alanine aminotransferase (ALAT) (*p* = 0.36), and γ-glutamyltransferase (γGT) (*p* = 0.76), did not show significant differences between the two groups. The mean ASAT was 111.37 ± 114.27 U/L in the HCV-Antibody+ group and 66.20 ± 31.65 U/L in the HCV-RNA+ group. For ALAT, the mean was 98.86 ± 127.56 U/l in the HCV-Antibody+ group and 42.60 ± 25.47 U/L in the HCV-RNA+ group. γGT levels were 61.38 ± 66.58 U/L in the HCV-Antibody+ group and 73.20 ± 54.43 U/L in the HCV-RNA+ group.

**Figure 1:**
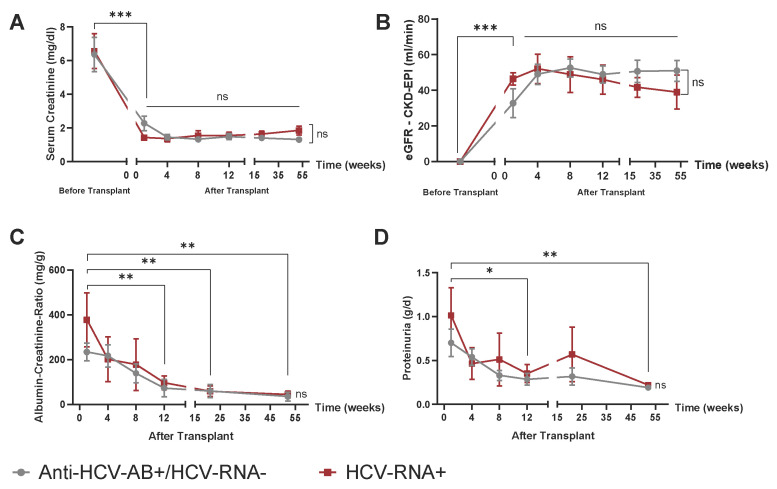
Kidney function before and after transplantation of a deceased donor kidney from Anti-HCV-AB+ vs. HCV-RNA+ donors. (**A**) Serum creatinine before and up to 52 weeks after transplantation with corresponding eGFR using the CKD-EPI formula. (**B**) Parameters for 24 h urine collection. (**C**) Albuminuria shown as albumin–creatinine ratio (mg/g). (**D**) Proteinuria (g protein/day). Mixed-effects analysis with multiple comparisons (time effects) as well as multiple *t*-tests (group differences), where *n* = 5 HCV-RNA+ vs. 8 Anti-HCV-AB+/HCV-RNA−. * *p*  <  0.05, ** *p*  <  0.01, *** *p*  <  0.001, ns = not significant.

Transplant function was stable in both groups, as indicated by serum creatinine levels at different time points starting one week after transplantation (2.2 ± 1.2 mg/dL in HCV-Antibody+/RNA− patients and 1.4 ± 0.3 mg/dL in HCV-RNA+ patients, *p* = 0.16) up to 52 weeks after the transplant (1.3 ± 0.4 mg/dL in HCV-Antibody+/RNA− patients and 1.8 ± 0.5 mg/dL in HCV-RNA+ patients, *p* = 0.08) (Figure 1A). Neither serum creatinine nor estimated GFR (Figure 1B) showed significant differences between the HCV-Antibody+/RNA− and HCV-RNA+ groups within the first 12 months post-transplant. Additionally, proteinuria did not differ between the two groups. This was evaluated using both the albumin-to-creatinine ratio (Figure 1C) and total protein excretion in g/day from 24 h urine collections (Figure 1D).

**Figure 2:**
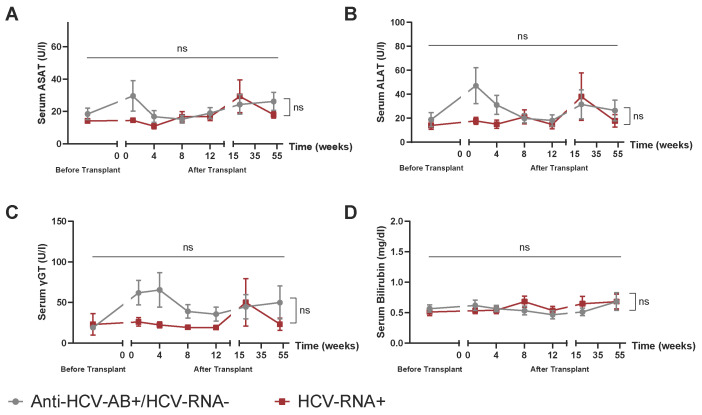
Hepatic outcome after transplantation of a deceased donor kidney from Anti-HCV AB+ vs. HCV-RNA+ donors. Serum levels of aspartate aminotransferase (ASAT) (**A**), alanine aminotransferase (ALAT) (**B**), γ-glutamyltransferase (γGT) (**C**), and bilirubin (**D**) up to 52 weeks after transplantation. Mixed-effects analysis with multiple comparisons (time effects) as well as multiple *t*-tests (group differences), where *n* = 5 HCV-RNA+ vs. 8 Anti-HCV-AB+/HCV-RNA−. ns = not significant.

We assessed the safety of post-mortem kidney transplantation from HCV-Antibody+/RNA− and HCV-RNA+ donors. We analyzed standard serum liver function parameters, including ASAT (Figure 2A), ALAT (Figure 2B), γGT (Figure 2C), and bilirubin (Figure 2D). For none of these parameters, a significant increase was observed within the first 12 months post-transplantation. Additionally, no differences were found between the HCV-Antibody+/RNA− and HCV-RNA+ group regarding these liver function markers.

**Figure 3:**
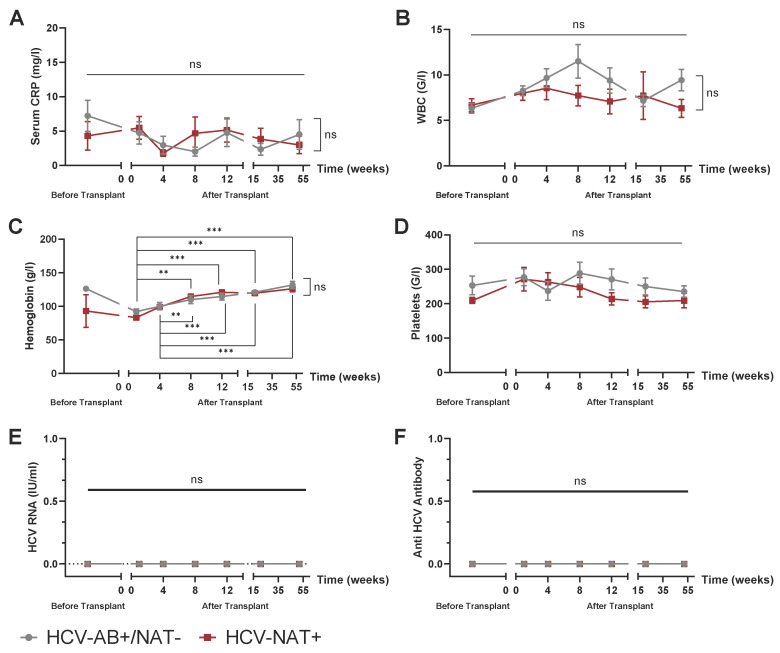
Longitudinal monitoring of systemic inflammation, hematologic parameters, and HCV markers in HCV-RNA+ and Anti-HCV AB+/HCV-RNA− kidney transplant recipients. Systemic inflammation, reflected by C-reactive protein (CRP) (**A**) and leukocyte counts (**B**). (**C**) Hemoglobin and platelet count (**D**). HCV-RNA (**E**) and Anti-HCV antibodies (**F**) during monthly follow-up visits within the first three months post-transplant or at the follow-up assessments at weeks 21 and 52. Mixed-effects analysis with multiple comparisons (time effects) as well as multiple *t*-tests (group differences), where *n* = 5 HCV-RNA+ vs. 8 Anti-HCV-AB+/HCV-RNA−. ** *p*  <  0.01, *** *p*  <  0.001, ns = not significant.

Systemic inflammation, as reflected by C-reactive protein (CRP, Figure 3A) and leukocyte counts (Figure 3B), did not differ between the two groups. Hemoglobin levels showed a consistent increase over the first year post-transplant in both HCV-Antibody+/RNA− and HCV-RNA+ donor kidney recipients (Figure 3C), while platelet counts remained stable throughout the study period (Figure 3D), with no significant differences observed at any time point. Importantly, no patients in either group tested positive for HCV-RNA during monthly follow-up visits within the first three months post-transplant or at the follow-up assessments at weeks 21 and 52 (Figure 3E). Additionally, none of the kidney recipients, regardless of donor HCV status, developed anti-HCV antibodies during the first year following transplantation (Figure 3F).

## 4. Discussion

Our retrospective single-center analysis evaluated the safety of transplanting kidneys from five HCV-RNA+ deceased donors into HCV-RNA− recipients. Pan-genotypic antiviral therapy (Glecaprevir/Pibrentasvir) was initiated on the day of kidney transplantation and continued for 12 weeks. The results were compared to eight patients who received kidneys from HCV-Antibody+/RNA− donors. We report stable transplant function in both groups without incidents of delayed graft function. Moreover, antiviral treatment was well tolerated as we did not observe any safety concerns that we retrospectively attributed to HCV viremia or antiviral therapy. HCV-RNA was neither detected in the HCV-Antibody+/RNA− nor the HCV-RNA+ group in the follow-up period of 52 weeks, supporting a preemptive antiviral therapy for transplantation kidneys from HCV RNA+ donors.

To increase the availability of organs and with safe and effective treatments at hand, HCV-RNA+ kidneys were transplanted into HCV-RNA− recipients. Several prospective trials have established that this is a safe practice [5,14,16,17]. In a prospective multicenter study, 30 selected HCV-RNA− patients received a kidney from a deceased HCV-RNA+ donor followed by an 8-week course of antiviral treatment. SVR could be achieved in 100% of cases for the first 12 months (SVR12), and graft function was excellent. Moreover, there were no serious adverse events related to viremia or antiviral treatment. Patients were treated with the pan-genotypic drug Glecaprevir/Pibrentasvir, which obviated the necessity of genotyping [18].

Outside of clinical trials, real-life data show that in most centers, DAAs are administered once viremia is detected and insurance approves antiviral therapies, which consequently delays treatment and carries the risk of clinical HCV infection. In a retrospective cohort study of 53 HCV-RNA− patients who received HCV-RNA+ kidneys, the median time between transplantation and DAA treatment initiation was 76 days. All patients achieved SVR12, and graft function was satisfactory. However, authors observed complications that might be attributable to HCV infection such as high rates of BK and CMV viremia [13]. By implementing the antiviral treatment directly after transplantation without waiting for viremia, the standard operating procedures at our center minimize the risk of unnecessary and potentially harmful HCV viral transmission to the patient. While current data are not conclusive [14], there have been reports about an increase in acute T cell-mediated rejections associated with the late initiation of treatment [13,19]. Fibrosing cholestatic hepatitis as a major complication of HCV infection occurred when there had been a delay in the initiation of antiviral treatment of more than 70 days [13,14,20], and this is most likely preventable by prompt antiviral treatment [20]. While more studies are warranted to determine which adverse events are directly related to HCV viremia, there is good evidence underlining that early clearance of infection is associated with fewer complications.

While many baseline characteristics such as mean recipient age or BMI were comparable in our cohort, the population in the clinical trials had a shorter duration of hemodialysis and did not include high-risk patients with relevant comorbidities [5,16,18]. Molnar et al. considered that these patients have especially small chances of receiving a kidney transplant in a timely manner and therefore could benefit most from expanding the donor pool with kidneys of HCV-RNA+ donors [13]. Similarly, Reese et al. also suggested offering HCV-RNA+ organs to high-risk patients with serious comorbidities [21]. Other researchers reported that waiting times prior to HCV-RNA+ kidney transplantation vary [14]. We observed significantly shorter times on the waiting list, reflected by time on dialysis, in patients receiving an organ from HCV-RNA+ donors as compared to organ donors who were HCV-Antibody+/RNA-. Compared to the overall national average waiting time in Germany [22], patients receiving transplants from HCV-RNA+ donors at our center experienced a waiting time that was more than two years shorter. Going forward, clear criteria should be established to determine eligible recipients for HCV-RNA+ kidney transplantation and to identify potential contraindications. As a life-prolonging procedure, it should not be withheld from patients when risks can be effectively minimized with appropriate treatment. In our study, there were no significant differences in recipients’ characteristics between organ recipients from HCV-RNA+ and HCV-Antibody+/HCV-RNA− donors. Although not meeting statistical significance, our data at least suggest that even longer ischemia times and a higher age in the recipient cohort of HCV-RNA+ donors did not result in a less favorable outcome. This might suggest that the general transplant population is suitable for receiving organs from HCV-viremic donors.

We did not observe HCV transmission by employing a preemptive DAA treatment of 12 weeks, as it has been described in the first clinical trials [5,16,17]. Despite this, the optimal length of treatment has not been defined yet, and shorter antiviral courses have been successful at achieving SVR12 [18,23,24,25]. In the US American healthcare system, DAA treatment has to be authorized by third-party payers, which is associated with an administrative effort and delayed treatment [19]. In the German healthcare systems, the costs of the 8-week medication regimen, with estimated expenses around EUR 30,000, are significant for insurance providers, which is similar to the situation in other countries [26]. Thus, preemptive treatment is often considered not to be feasible outside of clinical studies [27]. However, it is highly likely that antiviral medication will be necessary since HCV transmission rates in the context of transplantation are near 100% without DAA treatment [28]. Furthermore, there is good evidence that donor viremia correlates with recipient viremia [17], and as discussed above, early therapy is superior to delayed treatment. Furthermore, studies investigating cost-effectiveness do provide evidence that DAA treatment in general is cost-effective [26].

Fortunately, insurance approval was not necessary in our cohort prior to initiating treatment, and we can provide real-life data underlining the feasibility as well as the advantage for patients of preemptive antiviral treatment outside clinical trials. Our findings align with those of Franco et al., who also retrospectively evaluated the impact of preemptive DAA therapy in recipients of kidneys from HCV-viremic donors [29]. In their cohort of 41 recipients of HCV-RNA+ donor kidneys, they similarly observed no differences in graft function, patient survival, or graft survival. Notably, immediate DAA therapy effectively prevented or attenuated viral replication, further supporting the benefits of a preemptive approach. Given patient and insurer concerns, our data strongly support starting antiviral therapy immediately after transplantation to avoid harmful viremia. In our view, these benefits outweigh the treatment costs, especially considering the excellent prospects that kidney transplantation offers to recipients. The transplantation of kidneys from HCV-RNA+ donors, under the proper precautions, is a safe approach considering the current and supposedly ongoing organ shortage.

Kidney transplantations from HCV-Antibody+/HCV-RNA− donors into HCV-RNA− patients are considered safe and likely do not pose a risk of HCV-transmission [30,31]. This is underlined by our results as we did not detect HCV viremia in any patients receiving kidneys from HCV-Antibody+ donors. Nevertheless, NAT tests might be false negatives, or the infection might be recent in patients with risk behaviors. Therefore, regular testing for HCV-RNA in kidney recipients after transplantation would allow early treatment in the unlikely event of transmission.

Our study has several notable limitations. The relatively small sample size restricts the statistical power of our analysis, rendering the findings exploratory in nature and primarily hypothesis-generating. Currently, we can only provide short-term follow-up data, while long-term monitoring is ongoing. Additionally, the data presented reflect the experience of a single center, which inherently limits the external validity and generalizability of our results to other institutions or healthcare settings.

Nevertheless, we are pleased to contribute to the call to action outlined by Gordon et al. [14], who emphasized the importance of disseminating outcome data given the rarity of this clinical constellation and the resulting limited global treatment numbers. We agree that sharing even small data sets might help to advance the understanding of the optimal timing and duration of treatment, as well as the long-term clinical benefits and potential risks.

## 5. Conclusions

Our data provide real-world evidence on the beneficial effects of the immediate initiation of antiviral therapy without awaiting HCV-RNA positivity in the recipient. Our findings suggest that patients could benefit from this approach, as initiating treatment promptly may suppress any potential RNA replication before it becomes detectable. However, additional, larger multicenter trials are needed to validate these findings. Furthermore, our data from recipients of deceased kidney donations from donors with positive HCV antibodies but without detectable RNA suggest that the presence of HCV antibodies alone lacks clinical relevance and does not necessitate therapeutic intervention. However, implementing or maintaining serological surveillance in such cases may provide reassurance for both patients and healthcare providers.

## Figures and Tables

**Table 1:** Baseline characteristics of transplant recipients stratified by Hepatitis C status of the donor (Anti-HCV AB+/RNA− vs. HCV-RNA+). A *t*-test (age, body mass index, time on hemodialysis, ischemia time, and mismatches) or Fisher‘s exact test (all other parameters) was used. nd = No statistical testing due to small sample size.

	**All**	**HCV-AB+/RNA−**	**HCV-RNA+**	***p*** **-Value**
	*n* = 13	*n* = 8 (61.5%)	*n* = 5 (38.5%)	
Age at transplant, median (SD)	52.85 (11.4)	48.9 (12.5)	59.2 (4.3)	0.13
Body mass index (SD)	26.9 (3.1)	26.4 (2.8)	27.76 (3.4)	0.47
Hemodialysis (%)	13 (100)	8 (100)	5 (100)	0.99
Time on HD (months)	80 (37.4)	102 (27.3)	44.6 (20.0)	0.003
Residual diuresis (%)	8 (61.5)	6 (75.0)	2 (40.0)	0.29
Hypertension (%)	10 (76.9)	6 (75.0)	4 (80.0)	0.99
Diabetes (%)	2 (15.4)	1 (12.5)	1 (20.0)	0.99
Cause of End-Stage Kidney Disease				
Cystic disease (%)	3 (23.0)	3 (37.5)	0 (0)	nd
Diabetes (%)	2 (15.4)	1 (12.5)	1 (20.0)	nd
Hypertension (%)	1 (7.7)	0 (0)	1 (20.0)	nd
Congenital anomalies of the kidney and urinary tract (%)	1 (7.7)	1 (12.5)	0 (0)	nd
Focal segmental glomerulosclerosis (%)	1 (7.7)	0 (0)	1 (20.0)	nd
Rapidly progressive glomerulonephritis (%)	1 (7.7)	0 (0)	1 (20.0)	nd
Tubulointerstitial glomerulonephritis (%)	1 (7.7)	1 (12.5)	0 (0)	nd
Unknown/Not specified (%)	3 (23.0)	2 (25.0)	1 (20.0)	nd
Ischemia time (min)	837.2 (389.4)	750.4 (342.0)	976.2 (419.0)	0.35
Mismatches	3.5 (1.2)	3.6 (1.3)	3.4 (1.0)	0.76
CMV + recipient (%)	6 (46.2)	4 (50.0)	2 (40.0)	0.99
CMV + donor (%)	7 (53.8)	3 (37.5)	4 (80.0)	0.27
Antiviral Treatment with Glecaprevir/Pibrentasvir	5 (38.5)	0 (0)	5 (100)	0.001
Immunosuppressive Therapy				
Ciclosporin A	4 (30.1)	4 (50)	0 (0)	0.27
Tacrolimus	9 (69.2)	4 (50)	5 (100)	0.27
Mycophenolate mofetil	13 (100)	8 (100)	5 (100)	0.99
Prednisolone	13 (100)	8 (100)	5 (100)	0.99
Basiliximab	10 (76.9)	5 (38.5)	5 (100)	0.23

**Table 2:** Characteristics of deceased kidney donors stratified by Hepatitis C status of the donor (Anti-HCV-Antibody+ vs. HCV-RNA+ groups). Fisher’s exact test (female sex) or a *t*-test (all other parameters) was used.

	**All**	**HCV-AB+/RNA−**	**HCV-RNA+**	***p*** **-Value**
	*n* = 13	*n* = 8 (61.5%)	*n* = 5 (38.5%)	
Female sex (%)	7 (53.84)	5 (62.50)	2 (40.00)	0.59
Age at donation, median (SD)	48.92 (11.65)	44.25 (11.22)	56.40 (9.72)	0.09
Creatinine (mg/dL)	1.00 (0.56)	1.09 (0.68)	0.86 (0.28)	0.32
Bilirubin, total (mg/dL)	0.52 (0.43)	0.37 (0.15)	0.78 (0.56)	0.13
ASAT (U/L)	94.00 (86.06)	111.37 (114.27)	66.20 (31.65)	0.42
ALAT (U/L)	77.23 (95.45)	98.86 (127.56)	42.60 (25.47)	0.36
γGT (U/L)	65.92 (59.3)	61.375 (66.58)	73.20 (54.43)	0.76

## Data Availability

All data are presented in the manuscript and its accompanying files.

## Abbreviations

The following abbreviations are used in this manuscript:

ALAT	Alanine aminotransferase
ASAT	Aspartate aminotransferase
BMI	Body mass index
CMV	Cytomegalovirus
CRP	C-reactive protein
DAA	Direct antiviral agent
eGFR	Estimated glomerular filtration rate
HCV	Hepatitis C virus
KRT	Kidney replacement therapy
NAT	Nucleic acid testing
RNA	Ribonucleic acid
SVR	Sustained virological response
γGT	γ-Glutamyltransferase
